# Deciphering everyday meaning-making with Gramsci

**DOI:** 10.1007/s10624-022-09658-5

**Published:** 2022-06-01

**Authors:** Annika Lems

**Affiliations:** Max Planck Institute for Social Anthropology, Halle/Saale, Germany

**Keywords:** Gramsci, The everyday, Phenomenological anthropology, Alpine ethnography, Anti-cosmopolitanism

## Abstract

In this article, I take the principle underwriting Gramsci’s philosophy of praxis that ‘all men are philosophers’, as a point of departure to interrogate the anti-cosmopolitan everyday conceptions of the world I encountered during my fieldwork in an Austrian Alpine village in the midst of the Corona pandemic. In an attempt to understand the social and political force of such vernacular reasonings, I map the contours of a critical phenomenology of common sense. Following Gramsci’s lead, I reiterate that philosophical ideas uttered by the ‘man and woman in the street’ should be taken seriously by intellectuals. I argue that the moral and political judgements they contain do not just offer a unique basis for analysing the ways ideologies are rooted in the everyday, but also for tracing the intellectual currents underlying sedimented, exclusionary conceptions of belonging. In doing so, Gramsci’s philosophy of praxis enables phenomenologically oriented anthropologists to move beyond de-historicised and romanticised depictions of the everyday whilst keeping their focus on everyday acts of meaning-making. By analysing the anti-cosmopolitan common sense ideas I came across through a Gramscian lens, I suggest that his work can form a key avenue for deciphering the social, historical and intellectual currents propelling societal change.

## The biggest piss-take in the universe

*Every philosophical current leaves behind it a sediment of “common sense”: this is the document of its historical effectiveness. Common sense is not something rigid and immobile but is continually transforming itself, enriching itself with scientific ideas and with philosophical opinions which have entered ordinary life. “Common sense” is the folklore of philosophy, and is always half-way between folklore properly speaking and the philosophy, science and economics of the specialists.* (Gramsci 1971: 326)

In the fall of 2020, as I was trying to come to grips with the avalanche of anti-Corona stories flooding my home village in the Nock mountains (*Nockberge*) in the Austrian state of Carinthia, I kept returning to these words by Antonio Gramsci. My initial attempt to embark on an ethnography at/of home had been made impossible by the sudden outbreak of the COVID-19 pandemic in March 2020 and the national lockdown this led to in Austria. Because the rural area in the southernmost part of Austria where the municipality is located had emerged unscathed from the first wave, I had been able to pick up fieldwork again in the summer months, when the virus had retreated. However, by the autumn, this volatile situation started to change for the worse. As Carinthia was facing an exponential growth of infections, my research plans became increasingly difficult to sustain. This difficulty was amplified by the fact that a great deal of the people I interacted with rejected protection measures such as wearing a face mask, meeting outdoors or keeping a safe physical distance. Even though the Corona cases in the region had doubled within a short timeframe, and stories about neighbours, friends and relatives fallen ill with the virus had brought the reality of the pandemic closer to home, many, if not most, of the people I talked to dismissed the severity of the situation. There was a distinct sense of refusal in the air. Wherever I went, I was confronted with an unstoppable tide of opinions, stories and rumours that fundamentally questioned the validity and truthfulness of expert knowledge and the mainstream media.

Upon entering the village pub wearing a face mask, Anton, the forty-five-year-old owner, laughed at me. ‘No need for that rag here’, he said, pointing at my covered face. Even though the government had made the wearing of face masks in bars and restaurants mandatory, Anton was proud to explain that he did not abide by the rules. For him, Corona was the ‘biggest piss-take in the universe’. A few days later, when I had a chat with Tom, an electrician in his early thirties, he told me that ‘they [the government] can kiss my ass’ and that he would not follow any of the rules implemented to curb the spread of the virus. He was convinced that the Corona pandemic was just another way politicians tried to gain control over the lives of ordinary people like him. ‘I can already tell you who will be the winner out of all this, and it’s not going to be the supermarket cashiers’, he said, hinting at the government’s broken promise to reward supermarket workers for keeping up the nation’s daily food supplies when most other people were working from the safety of their homes. Even the local acupuncturist, a highly educated man in his fifties with a great sensitivity for health-related issues, encouraged me to take off my face mask when I went to see him—in spite of the poster pinned to the front door reminding patients to abide by the government regulations. In Jürgen’s opinion, the Austrian government was intentionally fabricating panic by locking everybody inside and forcing people to wear masks. He believed that face masks were causing more damage to the lungs and airways than an infection with COVID-19 could ever achieve. As the conversation continued, it became clear that Jürgen’s stance on Corona was not stable. He explained to me that the mainstream media refused to write about it, but that he knew for a fact that the hundreds of Corona patients treated in hospitals across the country were predominantly migrants. He said that because Muslim migrants had failed to ‘integrate’ properly, they did not follow the rules the government had introduced to reduce the spread of the virus. Even though he himself disobeyed the rules, Jürgen was confident that the rising infection rate was mainly thanks to migrants’ unwillingness to adjust to ‘our’ way of doing things.

While the newspapers were full of frightening reports about hospitals being stretched to the limit, the conversations I had with many villagers revealed a parallel universe of reasoning. The theories they shared with me took on different shapes and forms. As my conversation with Jürgen depicts, they were often contradictory, fragmentary and disjointed. What these everyday theories all had in common, however, was that they were marked by a commonly shared sense that the Austrian government was working against its own people. When I asked Anton, the pub owner, why he believed that the pandemic was a piss-take, he went into a long-winded monologue. He was convinced that politicians and global elites had fabricated the narrative of the pandemic to ruin local small businesses and replace Austrian workers with cheap foreign labour—a theory that reappeared in different forms and variations throughout my fieldwork. Recounting his experience from the first national lockdown, he said that he had been forced to pause his side gig as a bricklayer, his main source of income in the winter months when tourism came to a halt and the pub did not yield enough. ‘During the first lockdown they closed all the borders’, Anton said. ‘Me and my workmates couldn’t cross into Germany to get to the construction sites. But who was allowed to keep crossing borders as they pleased? – The black guys!’ Noticing the doubtful look on my face, he tried to convince me by reciting testimonies circulating on social media channels by people who claimed to be living in the Austrian border town of Braunau and had witnessed busloads of Africans being waved through by border personnel. Anton emphasised the dubious role of the government by arguing that not only were large groups of African migrants allowed to travel across Europe when everybody else was forced to stay at home, but that they were even officially escorted by the police. ‘They keep telling us that they won’t let in any more foreigners, but that’s bullshit. Look at all the black guys around. They let them in, and we know where this will lead to, right?’.

Anton’s story about the African migrants and his rhetorical question alluded to widely shared conspiracy theories about a *Bevölkerungsaustausch*, the replacement of the native, European population by migrants—meticulously planned and executed by left-leaning global elites who had successfully infiltrated politics. His conviction that governments across the world had invented the pandemic so that powerful industrialists could profit from the shattered economy, linked into ‘great reset’ theories that were circulating widely across right-wing social media networks and among the local population in Carinthia.^1^ They suggest that (mostly Jewish) world elites created the pandemic to devalue currencies and provoke a global economic crisis. In everyday conversations with Anton and other village inhabitants, these conspiratorial ideas appeared in vivid descriptions of Austrian politicians as weak, corrupt and preposterous, as mere puppets of the world’s wealthiest and most influential people. The powerful liberal elites pulling the strings from behind the scenes were described as cynical and heartless individuals who seized on the crisis situation to install a new, dystopic world order in which workers were replaced by robots and ordinary people forced to have chip cards implanted so that every move they made and every opinion they uttered would become traceable. When I asked Anton where he had learned about these things, whether there were websites or books he could refer me to, he laughed. If you were walking through life with a clear and critical mind, this knowledge was not hard to obtain, he noted. ‘It’s healthy human wit.’

The notion of healthy human wit (*gesunder Menschenverstand*) kept surfacing in my conversations with people living in the area. Whenever I asked where they had learned the commonly shared theories about the government trying to poison (through vaccinations), financially ruin and suppress (through the lockdown measures) or extinguish (through migration) its own population, the answer was clear: They knew it by making use of their healthy human wit.

## The abyss of healthy human wit

The ‘healthy human wit’ Anton and the people living in my home village referred to as the basis of their observations is the German equivalent of the English notion ‘common sense’. It is a sober, practical and realistic kind of knowledge that has grown out of life experience. G*esunder Menschenverstand* is inextricably linked to the idea of *die einfachen Leute* (ordinary people)—a figure that is diametrically opposite to that of the expert or intellectual (Dümling 2020). While expert knowledge is created in secluded, privileged spaces, healthy human wit is the product of the everyday, public realm. As hinted at by Anton, it is a natural slyness, an unspoiled kind of knowledge that cannot be learned from books, but that circulates on the streets—the domain of ordinary men and women.

Influenced by phenomenological and existentialist epistemologies, I have always been keen to develop my theorisation from people’s everyday engagements with the world (Lems 2018: 38ff). Propelled by the phenomenological leitmotif that scholars should direct their attention to the given, to the ‘things themselves’ (*die Sache an sich)* (Husserl 1970), as they appear to our consciousness prior to scientific abstraction, I have actively sought out people’s everyday meaning-making processes. Yet, the healthy human wit I was faced with (ironically!) on my own home turf left me feeling utterly puzzled and estranged. If I had so far unwittingly conceptualised the realm of vernacular knowledge production in positive terms—as a means for the marginalised, less powerful to voice their critique and generate ‘weapons of the week’ (Scott 1985)—the research in my home village confronted me with the irrefutable fact that everyday conceptions of the world are far more complex.

The healthy human wit I encountered in my home village seemed to emanate from a darker, more vexed place than I had been prepared for. It was not evocative of a progressive, more inclusive future, but of a regressive world order the contours of which resembled Austria’s Nazi past. The stigmatisation and scapegoating of migrants and Jews, the contempt for progressive intellectuals, the rejection of cosmopolitan world views and the deep-seated scepticism of liberal politics had been the main ingredients of the National Socialist’s success in the 1930s. The anti-Corona rumours and stories appeared to me as painful remnants of Carinthia’s past, when it formed one of the most important strongholds of Nazi support in the country (Elste 1997). It also echoed the long-lasting influence of the right-wing populist Austrian Freedom Party (FPÖ) on Carinthia’s socio-political landscape. Growing up as a teenager, the prevalence of these right-wing ideologies had propelled me to leave Carinthia as soon as I had finished school. Yet, if I took the phenomenological aim of developing theorisation from phenomena as they appeared in the realm of the everyday seriously, I could not bracket out acts of meaning-making that appalled me. As politically repulsive as some of the common sense ideas about the pandemic were, they still gave insights into people’s engagement with the world and the question of how they fitted in. Didier Fassin (2021: 128) points out that conspiratorial ideas and narratives should not too easily be written off as delusionary: ‘They are also indexes of social relationships, political tensions, cultural disquietude, and moral uneasiness’.

Confronted with the abyss of healthy human wit, Gramsci’s ponderings on common sense kept echoing in my mind. He wrote them against the backdrop of the massive crisis of parliamentary politics marking the European political landscape in the twentieth century, leading to a breakdown of the liberal architecture underwriting modern democracies, and ushering in the triumph of fascist ideologies (Martin 2015: 34). Unlike many other progressive intellectuals at the time, Gramsci did not denounce the supposed stupidity or backwardness of the ordinary men and women who formed the support base of the reactionary political order. Instead, he developed a new conceptual vocabulary to decipher the social grammars of the shared everyday opinions propelling political mobilisation. Gramsci was convinced that these ‘common sense’ conceptions of the world played a crucial role in the making and unmaking of the socio-political order (Crehan 2016: x). Because popular culture formed such an important terrain for political struggle, he insisted that it was the task of scholars to take the ‘spontaneous philosophy’ of the masses seriously (Gramsci 1971: 323). For Gramsci, the power of common sense was a critical reminder that ideologies were not solely an invention of intellectual elites that could be addressed by critiquing its philosophical fundaments, but that they were rooted and enacted in the everyday (Hall 1986: 20). Common sense was so powerful because it was able to identify ‘the exact cause’ of problems, ‘simple and to hand’, as Gramsci put it, without letting itself be distracted ‘by fancy quibbles and pseudo-profound, pseudo-scientific metaphysical mumbo-jumbo’ (Gramsci 1971: 348).

In what follows, I want to read the common sense theories about the COVID-19 pandemic I encountered in my home village with and alongside Gramsci. My aim is to map the contours of a phenomenology of common sense that does not just cast light on the complexity of everyday acts of meaning-making, but also on the politically charged nature of the everyday itself. In doing so, I am in conversation with the work of Veena Das (2020) and Eric Wolf (1982), two anthropological thinkers who, each in their own way, have stressed that a descent into the realm of the ordinary does not equal a descent into banality or ahistoricality. Albeit from very different conceptual angles, in their work of ethnographic theorisation, the everyday emerges as a crucial arena from where the complex relationship between the personal and the political, or the microcosm and macrocosm, can be observed and analysed. The lesson I take from these two thinkers is that the theories, tales and stories circulating in the Austrian hinterlands should not be written off as insignificant. The emergence of diametrically opposed claims to the truth can, to use Wolf’s (1999: 64) words, offer important empirical insights into ‘arguments and counterarguments over power and status’.

In what follows, I want to show that Gramsci’s immersive perspective and his focus on the spontaneous philosophies emerging from the domain of the everyday can form a key point of departure for a historically and politically informed phenomenological anthropology. It might seem counter-intuitive to bring phenomenological theorisations in conversation with one of the most important Marxist theorists. After all, phenomenological anthropologists have famously been criticised for failing to address the structural and historical conditions shaping social life (Lévi-Strauss 1973). Phenomenological anthropologists in turn have criticised dominant social and political theories for disconnecting structural questions from the particularity of people’s everyday experiences (Mattingly 2019: 419). The phenomenology of common sense I have in mind feeds off this ambivalent relationship between phenomenology and social theory. It deploys Gramsci’s work as a bridge between these two approaches of theorisation to develop an analytical lens that is able to do both—take seriously people’s everyday engagements with the world *and* the political and historical horizons against which these engagements take shape.

I will follow Gramsci’s lead and zoom in on the spontaneous, taken for granted ways the people in my home village questioned the socio-political order of things. By developing my theoretical takes from concrete and specific situations as they appear in the domain of the everyday, I can do justice to both, Gramsci’s aspiration to develop progressive social theory from a philosophy of praxis and the phenomenological aim to return to ‘things as they are’ (Jackson 1996). Revisiting Gramsci’s thoughts on common sense and his insistence that we must pay credit to the philosophical musings of people who are not philosophers, I will suggest that his work can be a key avenue for phenomenological anthropologists to address the everyday dynamics propelling societal change. Given that the *Prison Notebooks* are one of the most profound attempts to theorise the social power of reactionary world views, I believe that Gramsci’s work also shows the way for recent anthropological efforts to come to a deeper understanding of the processes of micro-mobilisation underlying the allure of the right (e.g. Hage 2017; Pasieka 2017).

## Tracing the historical inventory of common sense

One of the greatest obstacles to thinking contemporary appearances of common sense through a Gramscian lens is that his ideas often stubbornly refuse to be synthesised into widely applicable, timeless concepts. The scattered thoughts and notes making up the *Prison Notebooks* are notoriously difficult to read and interpret. This difficulty is fortified by Gramsci’s insistence on particularity, making it almost impossible to discern from his writings social patterns or theoretical rules that can be taken out of context. Given the fragmentary nature of his writings, most anthropologists attempting to apply Gramsci’s ideas to their own empirical material seek guidance in interpretations of his work. My own efforts corroborate this need for orientation. Besides Cate Crehan’s (2002; 2016) pathbreaking readings of the *Prison Notebooks*, Stuart Hall’s life-long engagement with Gramsci formed an important window into his world of ideas for me. Hall shows that Gramsci’s work can be used to make visible the historical conjunctures that lead to the emergence of contemporary formations of racism and ‘regressive modernisation’ (Hall 2017). He stresses that one of the main lessons scholars of race and ethnicity can take from Gramsci is his insistence on the importance of historical specificity (Hall 1986: 23). This also applies to everyday exclusionary ideas and practices. While there are general features of racism which are commonly shared, these features are shaped and transformed by the specificity of the contexts and environments in which they operate (ibid). It is precisely because of this insistence on the historicity of racist and anti-cosmopolitan ideas that Hall’s reading of Gramsci offers such an important point of orientation for my own efforts at deciphering the often xenophobic and deeply anti-liberal common sense permeating my home village. It allows keeping the focus on everyday practices whilst not overlooking the historical embeddedness of exclusionary acts of meaning-making.

To understand the shifting balance of power relations between the various social forces making up modern civil society, Gramsci drew on the comparison of historical case studies—for example, about the Italian Risorgimento, the French Revolution, Americanism or fascism. Because of their historical, national and political specificity, it would be problematic to analyse contemporary social phenomena through the same lens as the case studies that form the basis of his theorisation. Hall (1986: 6–7) suggests that to make more general use of Gramsci’s ideas, ‘they have to be delicately dis-interred from their concrete and specific historical embeddedness and transplanted into new soil with considerable care and patience’. For anthropologists to work with and alongside Gramsci’s concept of common sense, it is therefore essential to create this new soil by zooming in on the specificity of concrete ethnographic case studies of common sense. Such a focus on the particular is in accordance with phenomenological anthropologists’ tendency to emphasise the microcosm over the macrocosm (Jackson and Piette 2015: 5). Yet, a Gramscian approach yields more than a sharpened view on the micro-dimensions of social life. Gramsci insisted that critical thinking needed to start with a consciousness of one’s place in the world—an awareness that humans are a product of historical processes ‘which has deposited in you an infinity of traces, without leaving an inventory’ (Gramsci 1971: 324). He stressed that people’s everyday conceptions of the world are inextricably linked to the historical processes out of which they emerge (ibid: 325–269). The Gramscian phenomenology of common sense I have in mind can therefore not just be rooted in the social specificity of ethnographic case studies. It also needs to zoom in on the traces historical processes leave behind in people, forming and shaping their everyday conceptions of the world. Such a perspective reiterates John Cole’s and Eric Wolf’s (1999: xi) conviction that ‘anthropology cannot do without history’. In their path-breaking ethnography of two Alpine communities, they show that it is ‘only through an anthropologically informed historical account of the genesis and development of the forces impinging upon our social and cultural microcosms’ that we can come to a deeper understanding of ‘the ways in which these forces act upon each other in the present’ (Cole & Wolf 1999: xi). Translated to my own case study, this means that I need to expand my focus and look at the historical embeddedness of the common sense ideas I encountered in the Austrian mountain region where my fieldwork is located.

Healthy human wit—or *Alltagsverstand* (everyday knowledge), as German Gramsci scholars often describe it (Hirschfeld 2015)—is a crucial driver of political mobilisation in this part of Austria. This is not just the case in the present, with the global pandemic bringing about new forms of uncertainty. Throughout Carinthia’s modern political history, it pops up again and again. My home village is part of a municipality of about 4000 inhabitants who live spread over 18 small villages. The municipality is located in the Alpe-Adria region, an Alpine border triangle between Austria, Slovenia and Italy. It has thus always been linked into a world of movement and interconnection: The centuries-old trading routes between the Mediterranean and Central Europe criss-crossing this region are often described as symbolic of European integration (Valentin 1998; Moritsch 2001). Furthermore, tourism has formed a core source of income since the nineteenth century (Rogy 2002). The municipality’s idyllic position amidst the mountains and a lake turned it from a small, unimportant agricultural outpost into a popular *Luftkurort* (climatic spa), with three hundred thousand tourists streaming into the mountain villages every summer. Despite this cosmopolitan makeup, Carinthia has a long history of opposition against the decisions being ordered from the urban centres ‘above’, particularly the capital city of Vienna. Throughout the centuries, the region has been depicted as the rural, backward periphery, leading to fractious relationships with the changing centres of power (Valentin 1992; Rumpler and Fräss-Ehrfeld 2005). Cole and Wolf (1999: 42) point out that this culture of opposition can be traced back to the sixteenth century, when a rift started to appear between the flourishing upper-class cosmopolitan lifestyles characterising the Habsburg court of Vienna and the reality of peasants in the Austrian hinterlands whose struggles for self-determination had been brutally crushed. Historical accounts show this rift between Carinthia and Vienna to appear again and again, leading to self-depictions of the mountain villagers as proud and independent bearers of healthy human wit and fierce opponents to the changes imposed upon them by liberal city elites.

This sense of suspicion and outward hostility from the fringes towards the ruling centres continues to play an important role in the Austrian political landscape at large: Recent elections show an extreme cleavage between city and countryside, with urban centres voting for more progressive, cosmopolitan parties and the rural peripheries forming the stronghold of the far-right populist Freedom Party (FPÖ) (URL1). Carinthia is the state that represents this shift towards the right most dramatically: For over a decade, it formed the ideological bastion of notorious populist right-wing politician Jörg Haider (Ottomeyer 2010), and after a short break during which his party fell out of favour, it returned reinvigorated, with 33% of the Carinthian population voting for the FPÖ in the national elections in October 2017 (URL2). Despite a large-scale corruption scandal engulfing the FPÖ which led to re-elections and huge losses for the party in 2019, in Carinthia, it still achieved over 20% of the votes. The success of Jörg Haider and his successors is inextricably linked to their clever use of common sense narratives about corrupted urban political and intellectual elites, the misrepresentation of Austria’s Nazi past and the enforced denial of Austrian people’s ‘true’ cultural roots by the liberal paradigm. In this vein, Haider frequently spoke out ideas that had been banned by mainstream political parties in the post-war era but that kept circulating in the wider population. This includes his infamous praise for the Nazi regime’s employment policy, or his statement that Austrian soldiers fighting for the Wehrmacht were no perpetrators, ‘but at best victims’ (Zuser 1997: 25). When confronted with the moral outrage of moderate and progressive politicians, he would routinely retort by claiming that their obsession with political correctness and a lack of healthy human wit had led them to misinterpret his words. This strategy of political micro-mobilisation by tapping into vernacular anti-liberal theories was so successful that it came to be used by local politicians of all parties in Carinthia, including the left-leaning social democratic party. By making use of genres such as comics, jokes or caricatures, political actors continuously play with xenophobic, anti-semitic and anti-intellectual messages that appeal to the common sense of the ordinary ‘man and woman in the street’ (Wodak 2015). These common sense ideas are never consistent, but appear in countless different shapes and forms.

The conversations and interactions I observed in everyday village life revealed the slippery nature of common sense ideas. Tales about the legendary spirit of the late Jörg Haider were often also stories about the moral incorruptibility of Carinthian mountain people. Jokes about tabooed topics such as migrants or the sexualisation of women simultaneously transported a collectively shared sense of opposition against the silencing mechanisms of the urban upper classes towards ordinary people living in the countryside. And everyday theories about the fabrication of the pandemic usually also contained seeds of legitimate doubts about disaster capitalism and rising social inequality. Yet, while common sense ideas engendered important social critiques, this critical potential was not directed towards a more socially just future. More often than not this critique was channelled into conservative and nativist forms of protest that have been a recurring theme since the emergence of the Carinthia-Vienna rift in the sixteenth century.

Gramsci (1971: 419) noted that this theoretical slipperiness is characteristic for common sense. It is a ‘conception which, even in the brain of one individual, is fragmentary, incoherent and inconsequential’. The power of common sense is precisely that it is ‘a chaotic aggregate of disparate conceptions, and one can find there anything that one likes’ (ibid: 422). From a Gramscian perspective, it is the task of the scholar to create a sense of approximation to this landscape of fragmented ideas, stories and truths and to recover the systemic elements holding them together through careful empirical analysis (Crehan 2011: 282). The fundament holding together the various appearances of healthy human wit in Carinthia is the ambiguous relationship with Austria’s fascist past. While not always clearly articulated, they make use of everyday ideas, expressions and notions that tie into an established history of anti-cosmopolitanism.

## Historical figurations

Gramsci’s call to approach humans as historical beings and take seriously the ways the past enters and permeates the everyday echoes engagements with historicity and lived temporality in anthropology. While the historic turn in anthropology that developed from the 1980s onwards led to the acknowledgement that in non-Western cultural contexts people often deploy experiential, non-narrative ways of relating to the past (Hastrup 1992; Lambek 2002), in recent years, a growing number of anthropologists have argued that we need to go a step further and apply the same focus to European and North American societies (Hirsch & Stewart 2005; Rebel 2010; Hodges 2013). The prism of historicity allows anthropologists to look at history as a social practice: It enables them to focus on the everyday practices and ideas people deploy to actively make sense of and bestow meaning upon the past (Hodges, 2013: 479). Historicity, or ‘everyday history’, as phenomenologists prefer to call it (Carr 2014), is a helpful prism for approximating the social function of common sense ideas in Carinthia. At the same time, my case study calls into question its predominantly positive reading as a form of empowerment. Whilst the Carinthian example shows the importance of acknowledging the sense of agency the common sense ideas about Austria’s past creates in the people I worked with, it simultaneously shows the need to address the potentially violent effects these everyday meaning-making practices can have.

In her extensive work on right-wing common sense narratives in Austria, social linguist Ruth Wodak has shown how discursive practices of erasing or twisting uncomfortable historical details play into the power of reactionary political forces whose very success is based on the ambivalence of blurring past and present, fiction and reality (Wodak 2015). Indeed, Austria’s official treatment of the Nazi-past is a prime example of the far-reaching political potential of everyday histories. Scholars have shown how the maintenance of a common sense of an *Opfermythus* (victim myth) allowed historical events to be twisted to such a degree that perpetrators were turned into victims and vice versa (Bischof & Pelinka 1997; Utgaard 2003). The common sense ideas I encountered in my home village during the pandemic therefore did not appear out of the blue. They tie into alternative theories about Austria’s past and place in the world that might be banned from official history books but continue to surface in the shape of healthy human wit.

While a Gramscian phenomenology of common sense needs to be able to identify recurring historical themes, it is important not to think of these processes in terms of the simplistic repetition of historical patterns. Hermann Rebel (2010) forcefully shows why such an approach is problematic. Leaning on Eric Wolf’s work, he urges anthropologists attempting to carve out the long duration of Nazism not to equate ‘figurations’ with ‘templates’ (Rebel 2010: 85). While a historical template implies a ‘definable form that is consciously imposed by historical actors to shape and give meaning to their actions’, a figuration goes beyond the idea of a static representation of history, instead capturing a dynamic and moving return of images from the past (Rebel 2010: 86). Rebel warns against deploying ‘always-already-present “templates” for repeated enactments’ (ibid). To gain a deeper understanding of the ways remnants of the Nazi past are woven into the cultural fabric of Carinthian mountain communities, it is therefore crucial to understand the complexity of the ways the past interacts in the present. Such an undertaking needs to be based in the ‘recognition of a structuration dependent on multiple, interwoven microhistories in everyday life where systemic (i.e., systematically figured) collapses and reintegrations occur all the time, at any given and, most often, privately and historically concealed moment’ (Rebel 2010: 88).

Common sense ideas are thus always linked to historical processes. They are, however, not derived from simplistic historical templates, but from people’s direct engagements with the historical nature of the world they find themselves thrown into. The temporal ambiguity of the healthy human wit I encountered in my home village proofs Gramsci’s point that humans are essentially historical beings. This idea is also stressed by phenomenological thinkers, who have emphasized that humans are never mere observers of the historical world, but inextricably intertwined with it (e.g. Heidegger 1962; Dilthey 1978). David Carr (2014: 162) notes that ‘to say that we are historical beings is not merely to say that we are *in* history, that we arrive on the scene and then disappear at a certain point in objective historical time’. From a phenomenological perspective, historicity is ‘a feature *of our awareness itself*’ (ibid, emphasis in original). The common sense ideas about the pandemic I encountered in my home village do therefore not just link into a social world that is shot through with history. Their ambiguous embeddedness in time is indicative of the historicity of human meaning-making itself.

## Heritage Clubs in the War of Positions

My conversations with Anton, Jürgen and Tom should not be written off as the isolated, extremist derailment of frustrated individuals. Their seemingly disjointed common sense ideas about the pandemic link into wider social practices of sense-making that are based on profound critiques of Europe’s liberal foundations and the hegemony of cosmopolitan ideas. The social reproduction of this everyday anti-cosmopolitanism is best captured in the activities of the municipality’s heritage clubs (*Traditionsvereine*). These clubs form some of the most crucial pillars of social life—not just in this rural area of Austria, but across the German-speaking world (Hüwelmeier 1997). The German word for club—*Verein*—already hints at the important social role they occupy. It comes from the verb *vereinen*, which means to unify or bring together. These associations (*Vereinswesen*) have a long and complex history in German-speaking countries and link into nineteenth century nationalist projects that were accompanied by the invention and performance of traditions (Eidson 1994). Simply put, *Vereine* are legally recorded associations of individuals who are united by a common interest or goal. On a more complex level, however, they can be seen as vernacular social institutions through which ideas about history, culture and belonging to place are established, negotiated and performed.

Gramsci reiterated the pivotal role of clubs in the political architecture of modern societies. He argued that hegemony was not solely sustained through the power of the state, but also through complex negotiation processes with the institutions of civil society, including voluntary associations (Gramsci 1971: 243; cf. Hall 1986: 18). In Gramsci’s reading, clubs form the ‘the “trenches” and the permanent fortifications of the front in the war of positions’ (ibid). Far from being apolitical or insignificant, social institutions like the heritage clubs in Carinthia form an everyday frontline where struggles over the hegemony of ideas are fought out. Yet, it would be misleading to assume that these struggles occur in spectacular, extraordinary ways. Like common sense, the war of positions is shaped by the slow grind of history and the erratic rhythms of the everyday.

To better understand the ways the *Vereine* shape local power relations and define common sense ideas, it is necessary to sketch a more detailed ethnographic picture of the communities where my fieldwork took place. Despite being home to only about 4000 residents, there are roughly fifty active clubs within the municipality. Almost every inhabitant is a member of one or more clubs. In a remote area that has experienced decades of economic downturn and out-migration, *Vereine* take over functions that are pivotal to the social survival of communities. The Nock mountains region has one of the highest unemployment rates in Austria (URL3). The outsourcing of local jobs to low-cost countries over the last decades has initiated a large movement away from the villages to cities. This development has in turn provoked the slow but steady dismantling of key infrastructure in rural areas such as public transport, shops, schools and communal spaces, leading to their increased isolation. In my home village, these processes of decline are painfully obvious: While three decades ago there were three pubs, a grocery store, a bar and a bank, all of them except for one pub have disappeared since. Where public busses used to connect the village to the district capital every hour, they now only frequent twice a day. The clubs therefore occupy a vital role in the municipality. They form the most important spaces for people to meet and socialise, and the activities they organise (such as concerts, exhibitions, parties and competitions) strengthen cohesion both within the club and the larger community.

The club that Anton is also involved in, the *Heimatverein* (homeland club), demonstrates the crucial role clubs occupy in a social landscape that is marked by decline and neglect. Founded in the 1980s, the *Verein* aims to represent the cultural and historical roots of the people living in the municipality. The club’s main focus has been on the maintenance of a *Heimatmuseum* which is run entirely by amateur historians—local farmers and residents who have taken an interest in collecting historical objects and stories from the past. During the pandemic, the club initiated a project that was designed to respond to the social and economic pressures impacting upon the community. The *Verein* established a café in the museum which is run by club members who work there on a voluntary basis. The café is opened three days a week and also houses a small self-service store selling produce by farmers from the surrounding villages. The project is an attempt to revive social life in the village, encourage people to buy regional produce and strengthen a local sense of belonging. The café and store were welcomed enthusiastically by village inhabitants. Throughout the summer months of 2021, I was able to observe the remarkable transformation of the formerly abandoned and decaying space of the *Heimatmuseum* into a vibrant social and cultural hub.

For a rural community that has been plagued by decades of out-migration and dwindling infrastructure, the café represented an important place for harbouring a sense of cross-village solidarity. But it also acted as a laboratory for the creation and negotiation of common sense ideas about heritage, history and belonging to place. This was most strongly reflected in conversations about the club’s name. The notion of *Heimat* is heavily contested in the German-speaking world. It captures various ideas, including home, homeland or belonging and assumes a close connection between place and people, seeing them as historically and spiritually intertwined (Dickinson 2010: 582). Because of the abuse of the *Heimat* idea by the Nazis who suggested a direct a link between pristine Alpine landscapes and an ‘Arian’ national spirit, the use of the term has come under fire. In the past decades, many heritage clubs in Austria that were founded in the orbit of the nineteenth century *Heimat* movement (*Heimatbewegung*) have therefore changed their names. The members of the *Heimatverein* in my home village were highly critical of such acts of renaming. This was a crucial topic of debate, dominating many of the club gatherings. Based on common sense ideas about Austria’s postwar era and the pressure emanating from liberal elites to overwrite ordinary people’s ‘true’ cultural identities, club members fiercely defended their decision to keep the name. One core idea underwriting these discussions was that the local inhabitants’ special relationship to place needed to be protected. Mirroring Anton’s statements about African migrants attempting to seize on the pandemic moment to replace locally rooted lifestyles, club members voiced an urgent need to act against the soulless and rootless ‘multikulti’ (multicultural) paradigm propagated by city people. They saw the preservation of the populations’ natural or ‘indigenous’ cultural connection to the land as their core task. Yet, whilst aiming to preserve traditions, their actions were driven by exclusionary everyday conceptions of the world: Resembling the Nazi use of *Heimat*, they aimed to defend blood and soil from the socio-cultural infiltration of outsiders or from the spread of cosmopolitan ideals threatening to destroy their cultural ties.

The social reproduction of these exclusionary common sense ideas of belonging is also captured by the municipality’s most popular heritage club, the *Bürgergarde* (civil guards). Membership is restricted to men only, and the club has a strong influence on political decision-making in the village and the area at large. Members of the *Verein* trace its roots back to the fifteenth century, when the region was under attack by the Ottoman Empire, and local farmers organised themselves to protect the village from foreign occupation. While this version of historical events is the dominant narrative circulating about the club within the municipality, history books paint a less heroic picture. Rather than reproducing common sense ideas about the local farmers’ intrinsic relationship to the land or their glorious victory over foreign intruders, they speak of the centuries-long oppression of the farmers living in the mountain villages. In this version of the past, the *Bürgergarde* did not just grow out of the farmers’ patriotic fight against the Ottoman army, but was also the product of attempts by the clerical elite to control and crush peasant opposition. This history of exploitation and social unrest has been erased from common sense narratives about the club. Instead, club members and village inhabitants alike identify with its role as a defender of the community’s cultural integrity.

Anti-cosmopolitan historical figurations appear again and again in the club’s everyday actions and interactions. The *Bürgergarde* forms an integral part of community life in the municipality. During the most important village festivities, members of the club dress up in military uniforms and recreate the historical battle against the ‘Turks’. Given that Turkish migrants form some of the most politically charged public figures in Austria, the reenactment of their defeat signifies the exclusionary and territorialising undertone of the common sense underwriting the club’s actions. The club also plays an important role in caring for the *Kriegerdenkmäler* in the municipality— the memorials celebrating the villagers who died fighting for the Wehrmacht during the second world war. Club members see it as their task to ensure that the memorial spaces always look tidy, decorating them with flowers and candles. On festive occasions, the club marches to the memorial dressed in military uniform and carrying historical guns, paying their respects to ancestors who died fighting for the *Heimat*. This practice of commemorating people who actively participated in Nazi war atrocities links into the victim myth mentioned above—a commonly shared sense that official reports about the country’s fascist past are one-sided and force people to suppress their true cultural identities.

## The liberal paradigm and its counter-hegemonic currents

The everyday theories about belonging and non-belonging, history and culture promoted by the heritage clubs show that the common sense ideas about the pandemic I encountered in my home village did not come out of an ahistorical or apolitical void. They tie into a genealogy of anti-cosmopolitan practices, which, to rephrase Gramsci, has deposited in people an infinity of traces—traces that surface in their everyday engagements with the world. The aim of a Gramscian phenomenology of common sense is not just to detect and analyse these traces. Gramsci’s work was driven by an interest in piecing together the wider historical and intellectual inventory they grew out of. Doing so is a crucial step in understanding the ‘relations of force’ that constitute the terrain for political and social struggle (Gramsci 1971: 176). Because every socio-political order is based on the exclusion or repression of another one, it is the task of social scientists to look at the unstable equilibria the relationships between various forces produce (Hall 1986: 14). Repressed historical formations do not simply disappear. They keep lingering under the surface, slowly grinding off the base of the existing order, always keeping alive the possibility of another form of hegemony (Mouffe 2005: 18). In this vein, the anti-cosmopolitan common sense in my home village carries a politically charged metatext: It signals that while the liberal paradigm might have emerged victorious from the war of positions marking Austria’s post-fascist era, its hegemony does not remain uncontested. It is continuously destabilised by intellectual currents that question its validity.

The intellectual history of Carinthia and its relationship to the wider world provides a complex picture of the relations of force rubbing against each other. In this war of positions, currents of anti-cosmopolitan thought form the antipode to intellectual currents that are driven by liberal and humanist ideals. Again, these anti-cosmopolitan ideas do not appear out of nowhere. The German-speaking world has a pronounced history of anti-cosmopolitan thought and practice. This has been studied by social theorist Isaiah Berlin (1997) who coined the term ‘counter-Enlightenment’ to describe the traditions of thought that arose in the late eighteenth and early nineteenth centuries in opposition to the Enlightenment. In this work, anti-enlightenment emerges as a distinct intellectual and cultural tradition in Europe, which is based on a critique of the liberal, cosmopolitan ideas proposed by Enlightenment thinkers. It found its strongest manifestation in romantic literature and thought, most prominently in the writings of Herder and Rousseau, but it also echoed through the work of poets such as Lord Byron or the music of Schumann, Tchaikovsky or Strauss. In this intellectual tradition, the notion of alienation (*Entfremdung*) occupies centre stage, as it emphasises the deep sense of uprooting that is believed to be the effect of the ‘emptiness of cosmopolitanism’ characterising liberal ideals (Holmes 2000: 7; Jaeggi 2005). The idea that the hegemony of liberal philosophical currents is a form of cultural regress can also be found in the work of key German philosophers who are frequently used as the basis for contemporary right-wing ideologies (Beiner 2018). One of them is Friedrich Nietzsche, who directed his writings against the state of ‘horizonlessness’ he believed to be the outcome of liberal hegemony. In his view, the liberal paradigm had banished all meaningful horizons, leading to existential anguish, vertigo and homesickness (Nietzsche 1954; Beiner 2018: 35). Similarly, Heidegger’s oeuvre is marked by a pronounced critique of the ‘mere cosmopolitanism’ (*das bloße Weltbürgertum*) characterising the work of humanist thinkers such as Goethe (Heidegger 2000; cf. Beiner 2018: 91). Echoing the German poet Hölderlin, he believed that modern liberalism enforced cosmopolitan ideals upon ordinary people, thereby creating an existential sense of homelessness (Beiner 2018: 92–93).

The German-speaking Alpine region, and particularly the figure of the rural mountain peasant who is forced to break with his or her traditional cultural practices, played a crucial role in these anti-cosmopolitan ideas. The figure of the ‘lost’ Alpine peasant appears again and again in poetry, songs and philosophy to depict the cultural degradation that is the effect of liberalism. In these narratives, the sense of being displaced from one’s ‘natural territory’ or *Heimat* does not necessarily entail any form of physical dislocation. It is often purely figurative—a sense that liberal, cosmopolitan agendas force people to break with their enduring traditions and attachments to place in exchange for ‘rootless’ lifestyles (Holmes 2000: 7). These ideas did not remain limited to the domain of high culture and philosophy. Through the activities of heritage clubs and the *Heimat* movement, they found their way into the daily life of Carinthian mountain villages, thereby shaping people’s understanding of the world. The healthy human wit I encountered in my home village links into these wider intellectual currents of anti-cosmopolitan thought. It represents the inhabitants of rural Alpine villages as noble savages who hold their natural incorruptibility and down-to-earthness against the artificial, alienating culture of liberalism (Harvey 2012).

That the common sense ideas in my home village tap into an established inventory of anti-cosmopolitan thoughts proves Gramsci’s (1971: 165) point that ‘every philosophical current leaves behind a sediment of common sense’ and that the spontaneous philosophies emerging in the realm of the everyday feed off a complex dialogue between the knowledge intellectuals produce and vernacular meaning-making practices (Crehan 2016: xi). Common sense is therefore ‘strangely composite’ (Gramsci 1971: 324). It borrows from different intellectual traditions, combining many disparate ideas, from ‘Stone Age elements’ to modern scientific insights (ibid). Yet, the choice of philosophical ideas people base their everyday meaning-making on is not insignificant. While healthy human wit likes to present itself as ‘traditional wisdom or truth of the ages’ (Hall 1986: 20), it is a crucial socio-political terrain ‘on which more coherent ideologies and philosophies must content for mastery’ (ibid). Far from being apolitical, the realm of the everyday is a key arena of ideological struggle—a social space ‘which new conceptions of the world must take into account, contest and transform, if they are to shape the conceptions of the world of the masses and in that way become historically effective’ (ibid). A phenomenology of common sense that bases its epistemology on Gramsci therefore needs to dig much deeper than ‘things as they are’. To understand *why* things are the way they are (Desjarlais 1997: 25), it has to create a historicised angle and zoom in on the ways the social and political constantly push and shove into each other. Rather than treating the social and political as two separate realms, a Gramscian angle brings to the fore their inseparability. In doing so, it does not just cast new light on the political potential of people’s shared meaning-making practices. While keeping the focus on everyday life in its givenness, it works as a reminder not to overlook the inherently political nature of the everyday itself.

## By way of conclusion: De-romanticising everyday meaning-making

The contours of the phenomenology of common sense I have attempted to sketch in this article shows how far-reaching Gramsci’s ideas can be if anthropologists apply them to everyday meaning-making practices. It allows them to shed light on the political potential of people’s vernacular conceptions of the world and on the social and existential struggles underlying them. Such an angle can only work, however, if we treat Gramsci’s intellectual legacy with conceptual carefulness and empirical precision. Importantly, a Gramscian phenomenology of common sense never occurs in a temporal void. By treating humans as historical beings through and through, it brings to the fore the various intellectual currents and historic formations shaping everyday conceptions of the world.

In bringing this thought-experiment to a close, I want to emphasise one final element of Gramscis’ philosophy which needs to be part and parcel of any anthropological engagement with common sense. Besides shedding light on the social and political nature of everyday conceptions of the world, a Gramscian perspective also entails a critical reflection of the common sense ideas *about* common sense circulating within the social sciences. In anthropology, vernacular meaning-making practices—as, indeed, the realm of the everyday itself—are often treated in taken-for-granted ways. As Veena Das (2006: 6–7) notes, human relationships ‘require a repeated attention to the most ordinary of objects and events’, yet the very ordinariness of the everyday often makes it difficult for anthropologists to see what is before their eyes (Das 2020: 15). The pronounced desire by anthropologists to speak against dominant regimes of power (Ortner 2016) in the last four decades has brought about a fascination with vernacular forms of resistance or ‘agency’. It has led to a keen interest in everyday meaning-making practices that allow the marginalised, subaltern, ‘suffering subject’ (Robbins 2013) to break out of its muted role and formulate critiques of power. In these anthropological readings, everyday meaning-making has taken on a positive, and somewhat simplistic, slant, as a vernacularised expression of dissent against oppression and exploitation. In many ways, anthropologists’ common-sensical treatment of everyday meaning-making as a form of resistance of the oppressed might be able to tell us more about anthropologists’ urge to search out ‘pockets of hope’ (Kleist & Jansen 2016: 378) amidst a world of uncertainty than it does about everyday-meaning making practices per se. Rarely theorised (Das 2020; Jackson 2013), the realm of the everyday emerges from these debates as a fuzzy, non-descript and politically innocent social space in which the lives of ordinary women and men unfold. Yet, the exclusionary conceptions of the world circulating in my home village show that the realm of the everyday and the philosophical ideas emerging from it are complex social entities that cannot be understood without taking into account the historical and political horizons against which they take shape. They demonstrate that while common sense ideas can contain traces of oppositional world views, these views are often ‘crudely neophobe and conservative’ (Gramsci 1971: 423). Rather than turning away in disgust, a Gramscian perspective on common sense urges anthropologists to look closely at such everyday conceptions and ideas, no matter how politically difficult they might be.

The anti-Corona and anti-cosmopolitan healthy human wit I encountered in Carinthia shows the politically charged nature of common sense—how it derives its power from the integration of different, often contradictory ideological elements and manages to turn the everyday into a terrain for micro-mobilisation. Gaining a deeper understanding of this politically and affectively charged terrain is a crucially important task for contemporary anthropology. Throughout the past decades, there has been a wealth of research across the social science on right-wing party structures, strategies and leadership (Betz & Immerfall 1998); the longue durée of twentieth century authoritarian movements (Utgaard 2003; Neumann 2003); and the circulation of right-wing populist discourses and narratives fostering fear and resentment (Wodak 2015), as well as ethnographies of extremist sub-cultures such as neo-Nazis, Skinheads or religious fundamentalists (Shoshan 2016). While these studies give crucial insights into the various factors driving political radicalisation, not much is known about the lifeworlds of ordinary people supporting exclusionary political movements. They are part of a rapidly growing proportion of the population in liberal democratic societies who no longer want to adhere to the rules of the ‘liberal game’ (Illouz 2017: 49) and openly reject its core ideals of diversity and tolerance. A Gramscian phenomenology of common sense can help us move beyond simplistic portrayals of the people living in rural strongholds of right-wing parties such as my home village as an undifferentiated mass of backward, bigot, and uneducated consumers of extremist discourse. By zooming in on the ways their everyday meaning-making practices tie into counter-hegemonic currents of thought, it can show them as individuals and collectives with their own histories of vernacular ideas that need to be considered if social scientists want to understand the political fragmentation of the public domain that is taking hold.

Throughout his career, Gramsci was sceptical of progressive scholars’ tendency to exoticize the lifeworlds of the peasants or workers they studied. Instead, he opted for a ‘clear and unsentimental attitude’ that avoided ‘both romanticization and demonization’ (Crehan 2002: 14). A Gramscian reading of common sense demonstrates that everyday meaning-making practices should not be romanticised. While they carry the potential for a more socially just, progressive world order, they can also be the seeds for a darker, exclusionary future. The tide of anti-cosmopolitan, anti-liberal and anti-intellectual healthy human wit I was confronted with in my home village at the height of the Corona pandemic shows the political potential of the vernacular theories circulating in the everyday. Anthropologists have rightfully emphasised the significant social role of such theories as weapons of the weak (Scott 1985). Yet, I agree with Veena Das’s (1998) observation that they do not just form vehicles through which the masses are mobilised to redress moral wrongs: While everyday conceptions of the world have fuelled peasant uprisings and social revolutions, they have also mobilised spectacular crowds in support of fascist regimes. It is this double-edgedness of common sense that a Gramscian perspective allows anthropologists never to lose sight of. The everyday philosophies put forward by people like Anton carry important moments of critique against the inequality and existential alienation that are the outcome of processes of global capitalist transformation. But they also bring to life ‘structures of thought’ (Das 1998) that legitimise violence against people and groups marked as dangerous or threatening.

## Footnotes

1 Great reset conspiracy theories originate in an identically named book from 2011 about the revival of the economy after the world economic crisis in 2008 (Florida 2011). In May 2020, the World Economic Forum organised its annual conference under the same slogan. They picked up the title to explore how countries might recover from the economic damage caused by the pandemic. After Joe Biden won the US presidential elections, theories about the sinister nature of these plans and his involvement in it started to circulate on the Internet, mainly through right-wing social media networks.
